# SARS-CoV-2 Omicron (B.1.1.529) Variant: Corticosteroids Treatment/Respiratory Coinfection

**DOI:** 10.3389/fimmu.2022.856072

**Published:** 2022-03-03

**Authors:** Zeev Elkoshi

**Affiliations:** Research and Development Department, Taro Pharmaceutical Industries Ltd., Haifa, Israel

**Keywords:** COVID-19, SARS-CoV-2, Omicron variant, B.1.1.529, regulatory T cells, binary classification of chronic diseases, co-infection

In a recent publication, the binary model of chronic diseases was applied to SARS-CoV-2 infection (1). The efficiency of corticosteroids in the treatment of severe COVID-19 was explained by their ability to promote Treg expansion while sparing SARS-CoV-2 specific CD8^+^ T cells. In addition, the model deciphered the coinfection of several respiratory pathogens and severe COVID-19 disease afflicted by early variants of the SAS-CoV-2 virus. The high prevalence of these coinfections was explained by the pro-inflammatory and “low Treg” nature shared by respiratory pathogens and severe (“chronic”) COVID-19 triggered by these early variants.

Early assessment of the clinical severity of SARS-CoV-2 Omicron variant (B.1.1.529) in South Africa suggested lower odds of severe disease compared to the Delta variant (2). Another study conducted in South Africa pointed to a reduced mortality during the Omicron wave compared to earlier waves of COVID-19 (3). Similarly, a Canadian study comparing the Omicron and Delta variants presented a reduced severity with the Omicron variant (4). A cohort analysis with nested test negative design study, investigating Omicron severity in Scotland, concludes that “Omicron is associated with a two-thirds reduction in the risk of COVID-19 hospitalization when compared to Delta” (5). A US retrospective study comparing COVID-19 outcomes before and after the emergence of Omicron indicates a significantly less severe outcome during the Omicron wave (6). Corrected estimates adjusted for under-ascertainment of reinfection, demonstrate about 25% reduction in the probability of hospitalization of an unvaccinated person with no history of SARS-CoV-2 infection when Omicron is compared to the Delta variant (7, 8). Although the question of the relative severity of Omicron infection is still under discussion in the literature (9), Omicron seems milder than earlier variants (10). With the earlier variants of SARS-CoV-2, a severe disease was related to a surge of pro-inflammatory cytokines, the so called “cytokine storm” (11). This “chronic” high pro-inflammatory state correlated with low Treg levels (1). The seemingly lower intrinsic severity with Omicron may suggest a lower extent of cytokine storm with this variant. In line with this, the Omicron variant demonstrated attenuated lung infection in several rodent models (12, 13). In the overwhelming majority of patients, this variant of concern presumably triggers an acute immune reaction which eventually decays. Resolution of this acute inflammation results in low levels of pro-inflammatory cytokines, along with relatively high levels of regulatory T cells (1). Even if Omicron related inflammation persists, it is plausibly attenuated and may be considered a “high Treg” inflammation. Under this assumption, the binary model of chronic diseases (14) predicts: (a) for the vast majority of Omicron variant cases, corticosteroid treatment is not recommended (since corticosteroids are Treg promoters (1)); (b) corticosteroids may be effective in Omicron related severe disease *only if a surge in the blood levels of cytokines like IFN-γ, IL-1, IL-6, TNF-α or IL-10, has been observed*; (c) the frequency of coinfection with *Respiratory Syncytial Virus, influenza A virus*, *Influenza B virus*, *Parainfluenzae*, *Mycoplasma pneumoniae*, *Pseudomonas aeruginosa*, *Haemophilus influenza* or *Klebsiella pneumoniae* is expected to be lower compared to earlier variants [since all these pathogens induce “low Treg” reaction (1)];(d) in Omicron associated pneumonia, the frequency of coinfection with *Staphylococcus aureus*, *Streptococcus pneumonia*, and *Adenoviruses* is expected to increase since each of these three pathogens induces “high Treg” reaction (15–18). For the same reason, corticosteroids are not expected to be efficient in the treatment of pneumonia associated with these pathogens. A lower extent of respiratory coinfection possibly contributes to the observed lower intrinsic severity of Omicron, compared to Delta and other earlier variants of the SARS-CoV-2 virus.

## Author Contributions

The author confirms being the sole contributor of this work and has approved it for publication.

## Author Disclaimer

The views and opinions expressed, and/or conclusions drawn, in this article are those of the author and do not necessarily reflect those of Taro Pharmaceutical Industries Ltd., its affiliates, directors or employees.

## Conflict of Interest

Author ZE is employed by Taro Pharmaceutical Industries Ltd.

## Publisher’s Note

All claims expressed in this article are solely those of the authors and do not necessarily represent those of their affiliated organizations, or those of the publisher, the editors and the reviewers. Any product that may be evaluated in this article, or claim that may be made by its manufacturer, is not guaranteed or endorsed by the publisher.
